# Tinea Corporis-associated Erythroderma: Case Report and Review of Erythrodermic Patients with Chronic Dermatophyte Infection

**DOI:** 10.7759/cureus.7578

**Published:** 2020-04-07

**Authors:** Faraz Yousefian, Christopher Crowley, Hadas Skupsky, Antoanella Calame, Philip R Cohen

**Affiliations:** 1 Osteopathic Medicine, University of the Incarnate Word School of Osteopathic Medicine, San Antonio, USA; 2 Dermatology, San Diego Family Dermatology, National City, USA; 3 Dermatopathology, Compass Dermatopathology, San Diego, USA; 4 Dermatology, Compass Dermatopathology, San Diego, USA

**Keywords:** corporis, dermatitis, dermatophyte, erythroderma, exfoliative, hyphae, scaling, skin, tinea, trichophyton

## Abstract

Erythroderma presents as generalized skin redness. The features of a 39-year-old man who presented with erythroderma are described. His skin biopsy revealed hyphae in the stratum corneum, which established the diagnosis of tinea corporis. His erythroderma resolved following treatment with an oral antifungal agent. Several conditions can be associated with erythroderma. Common etiologies for erythroderma include medications, neoplasms, and papulosquamous disorders. Superficial dermatophyte-associated erythroderma is rare. However, although erythroderma caused by generalized superficial mycosis is infrequently encountered, tinea corporis should be included in the new-onset or chronic erythroderma. The detection of fungal hyphae in the stratum corneum of a biopsy of the erythrodermic skin can not only establish dermatophyte infection as the underlying cause of the individual’s erythroderma but also an alternative cause of erythroderma.

## Introduction

Erythroderma is also referred to as exfoliative dermatitis. It presents as diffuse redness of the skin. There are several potential etiologies for erythroderma [1-3].

Dermatophytes can be classified into three groups, including trichophyton, microsporum, and epidermophyton. They are capable of invading and multiplying in keratinized tissues, thereby leading to infection [4]. Tinea corporis is a superficial dermatophyte infection. It classically presents as an erythematous scaly plaque. However, rarely, tinea corporis may present as erythroderma [4-7]. 

A man who developed erythroderma-associated tinea corporis is described. The features of another erythrodermic individual with dermatophyte infection are also reviewed.

## Case presentation

A 39-year-old immunocompetent, human immunodeficiency virus seronegative, man presented with an exacerbation of a chronic condition, which had persisted for more than six years. His peeling, red skin was asymptomatic. However, he was concerned about recent skin changes and decided to seek medical attention. He had no personal or family history of either dermatitis or psoriasis; yet, his medical history was significant for both hypertension and heart failure. His current medications included amlodipine, clonidine, furosemide, and lisinopril.

**Figure 1:**
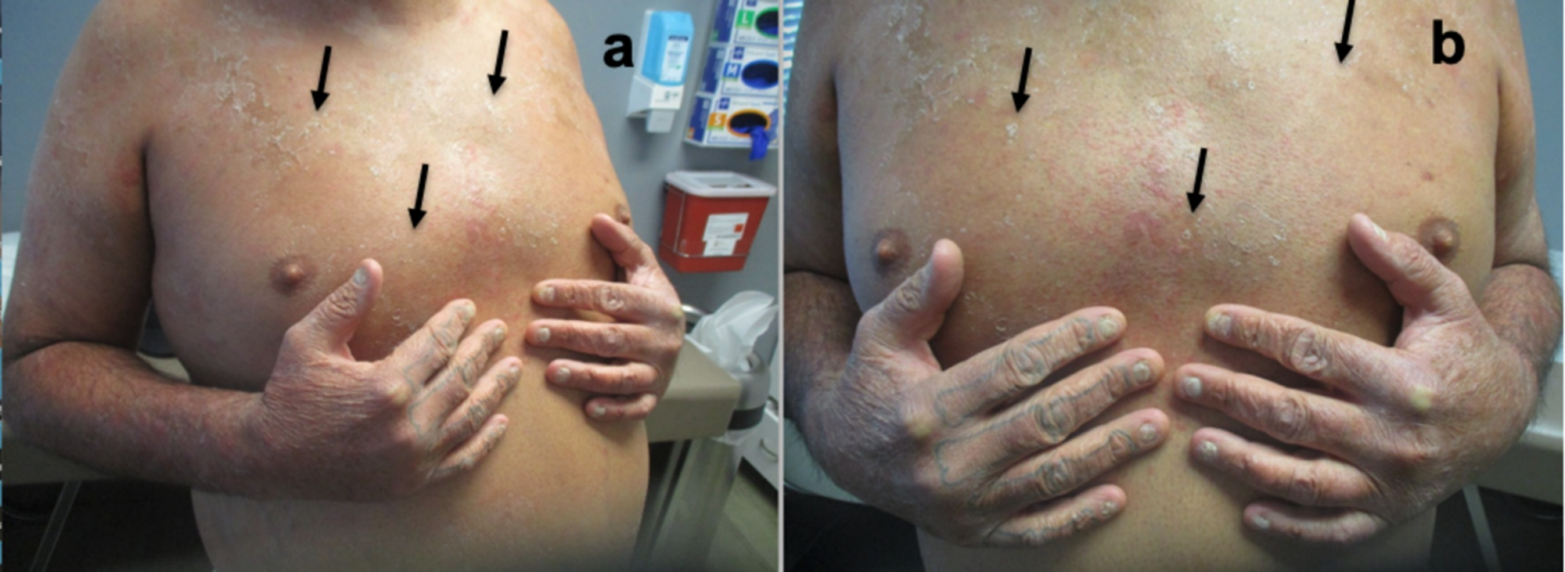
Clinical presentation of tinea corporis-associated erythroderma Distant (a) and closer (b) views of the chest, abdomen, and upper extremities of a 39-year-old man with generalized erythroderma presenting with diffuse redness of his skin and superficial desquamation (black arrows).

Cutaneous examination revealed generalized erythroderma and superficial desquamation of the skin; his trunk and extremities were predominantly involved. In addition, his fingernails were dystrophic and discolored (Figure 1).

**Figure 2:**
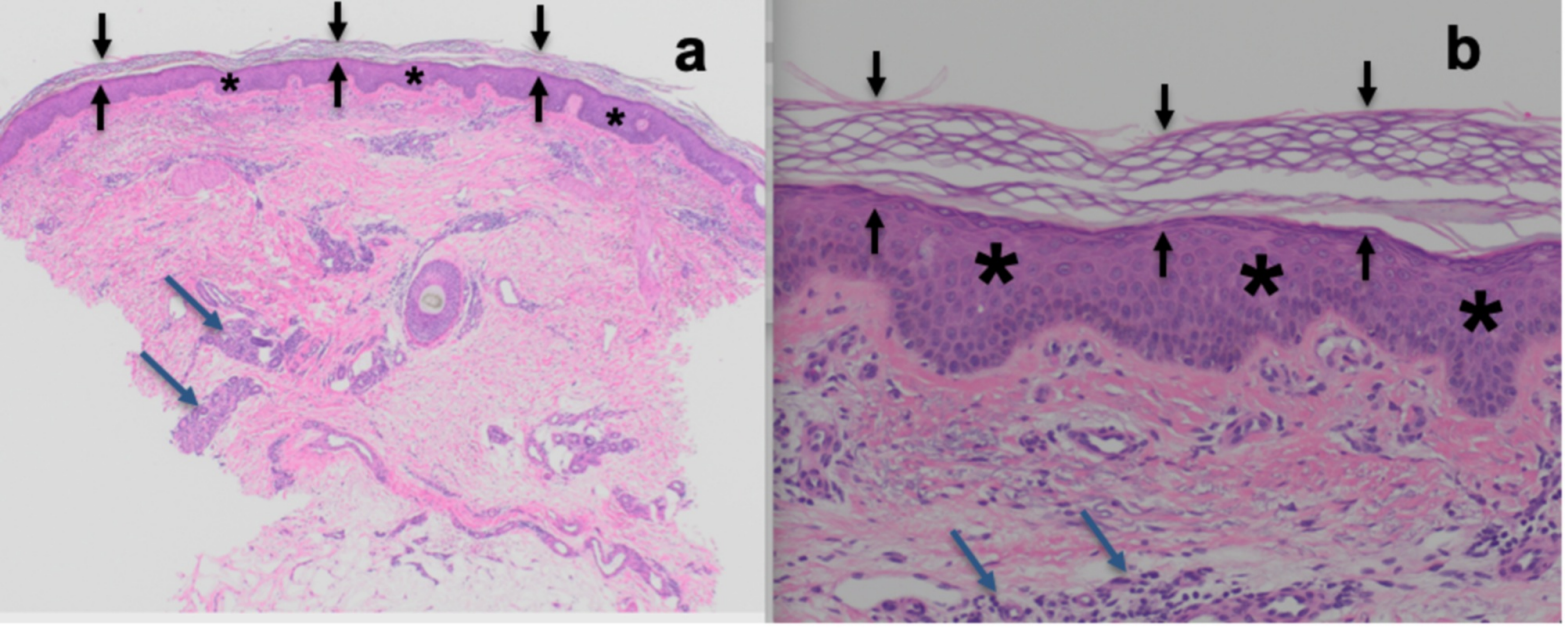
Microscopic findings on hematoxylin and eosin stained sections of tinea corporis-associated erythroderma Distant (a) and closer (b) views of a skin biopsy from the left forearm show basket-weave orthokeratosis of the stratum corneum (between black arrows), acanthosis of the epidermis (asterisk), and lymphohistiocytic inflammation in the dermis (blue arrows) (hematoxylin and eosin: a = x4, b = x20).

Microscopic examination of the hematoxylin and eosin-stained sections of the skin biopsy obtained from the left forearm showed mild acanthosis with overlying orthokeratosis; there was a lymphohistiocytic infiltrate in the dermis (Figure 2).

**Figure 3:**
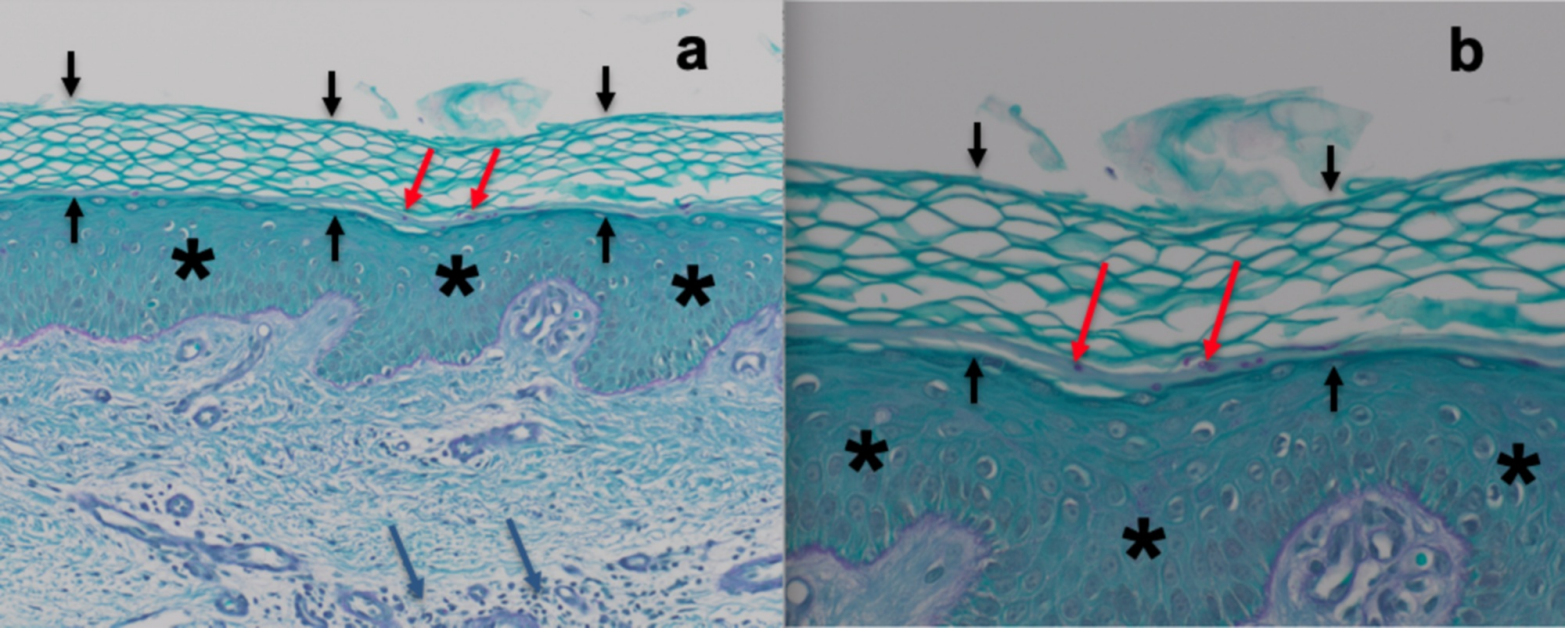
Microscopic findings on periodic acid-Schiff stained sections of tinea corporis-associated erythroderma Distant (a) and closer (b) views of the biopsy of erythrodermic skin not only show orthokeratosis (between black arrows), acanthosis (asterisk), and dermal inflammation (blue arrows), but also purple staining fungal hyphae, which appear as oval structures in the stratum corneum when they are cut in cross-sections (red arrows), confirming the diagnosis of dermatophyte infection (periodic acid-Schiff: a = x20, b = x40).

Periodic acid-Schiff (PAS) stain revealed PAS-positive hyphae in the stratum corneum, thereby confirming a diagnosis of tinea infection (Figure 3). 

Correlation of the clinical presentation and the pathology findings established a diagnosis of tinea corporis-associated erythroderma. Laboratory studies were performed. His complete blood cell counts and platelets were normal; however, his serum chemistries revealed an elevated blood urea nitrogen (45 mg/dl; normal = 7-25 mg/dl) and creatinine (2.27 mg/dl; normal = 0.60-1.35 mg/dl).

Prior to our evaluation, the patient had been prescribed betamethasone 0.05% cream and triamcinolone 0.1% ointment without improvement. Once the diagnosis of dermatophyte-related erythroderma was established, he received antifungal-targeted treatment consisting of both ketoconazole 2% shampoo and cream. The patient only achieved a partial improvement of his condition.

Systemic treatment was initiated; his liver function tests were normal. He received oral fluconazole 75 mg per week for six weeks. He experienced complete resolution of his erythroderma.

## Discussion

Erythroderma clinically presents as redness and scaling of the majority of the skin; pruritus may also be present. It is relatively uncommon; indeed, erythroderma only occurs in one out of every 100,000 people per year. Although erythroderma occurs in both men and women, the vast majority of erythrodermic patients are older men [1,8-10]. 

Erythroderma can arise from a variety of causes. Exacerbation of a preexisting inflammatory condition, such as psoriasis, is the most common etiology. Other potential causes of erythroderma include drug reactions and neoplasms (Table 1) [1-10].

**Table 1:** Common causes of erythroderma ^a^These include captopril, enalapril, and lisinopril. ^b^These include penicillin, streptomycin, and sulfones. ^c^These include carbamazepine, phenobarbital, and phenytoin. ^d^These include angiogenesis inhibitors, such as bevacizumab and kinase inhibitors such as imatinib. ^e^This includes hydroxychloroquine. ^f^This includes chlorpromazine. ^g^This includes isoniazid. ^h^This includes erythropoietin. ^i^These include piroxicam and sulfasalazine. ^j^These include esomeprazole, omeprazole, and pantoprazole. ^k^These include acitretin and isotretinoin. ^l^This includes allopurinol. ^m^These include cutaneous T-cell lymphoma (including Sezary syndrome), Hodgkin’s and non-Hodgkin’s lymphoma, and leukemia. ^n^These include colon cancer, hepatocellular carcinomas, lung cancer, and renal cell carcinoma.

Erythroderma etiologies
Eczematous disorders
Atopic dermatitis
Chronic actinic dermatitis
Contact dermatitis
Seborrheic dermatitis
Medications
Angiotensin-converting enzyme inhibitors^a^
Antibiotic^b^
Anticonvulsants^c^
Antineoplastics^d^
Antimalarials^e^
Antipsychotics^f^
Antituberculosis agents^g^
Colony-stimulating factors^h^
Nonsteroidal anti-inflammatory drugs^i^
Proton-pump inhibitors^j^
Retinoids^k^
Xanthine oxidase inhibitors^l^
Neoplasms
Hematopoietic dyscrasias^m^
Solid tumors^n^
Papulosquamous disorders
Dermatophyte infection
Ichthyosis
Lichen planus
Papuloerythroderma of Ofuji
Pityriasis rubra pilaris
Psoriasis
Sarcoidosis

Erythrodermic patients with chronic dermatophyte infection have been described in three clinical settings. One of these scenarios is similar to our patient: erythroderma that is a direct consequence of only the tinea corporis infection. The second situation of erythroderma-related chronic tinea includes individuals whose erythroderma is multifactorial; for example, their erythroderma may be secondary to not only a chronic dermatophyte infection but also other etiologies such as a paraneoplastic phenomenon or an ‘id reaction’. The third setting of erythroderma-associated chronic tinea occurs in patients with congenital disorders characterized by erythroderma who subsequently acquire a persistent dermatophyte infection.

Tinea corporis-associated erythroderma has previously been described in a 64-year old female nurse. She developed Trichophyton rubrum-induced erythroderma that affected her skin, hair, and nails. The investigators attributed her condition to several potential causes: her advanced age, the prolonged use of topical corticosteroid ointments, local infection, and the medications she handled while working [5].

Chronic dermatophyte infection was also described in a man with recurrent and persistent erythroderma. Initially, at age 20 years, he developed an exfoliative dermatitis (that was also referred to as either ‘a generalized weeping dermatitis’ or ‘a persisting generalized exudative eruption’). During a dermatitis episode, skin scrapings not only demonstrated fungus, but also cultured Epidermophyton floccosum; his skin cleared after three months of oral griseofulvin. However, during the following 14 years, he continued to experience additional episodes of erythroderma that did not respond to oral antifungal therapy. At age 34 years, he developed gastrointestinal symptoms in addition to his erythroderma; although fungus was found in his palms, feet, and nails, there was none on biopsies of erythrodermic skin. The systemic evaluation discovered a histiocytic lymphoma (reticulum cell sarcoma) of his stomach and distal esophagus; he experienced a complete recovery and remained well following esophagogastrectomy. The researchers speculated that his original erythroderma was dermatophyte-related, and his more recent erythroderma was either paraneoplastic from the lymphoma or an ‘id reaction’ from his fungal infection [6].

Generalized chronic tinea infection has also been observed in suberythrodermic patients with an underlying congenital disorder such as ichthyosiform erythroderma. A 41-year-old man had chronic suberythrodermic culture-confirmed Trichophyton violaceum of his hair and skin since the age of five years. His older sister, age 44 years, also had the identical clinical manifestation of Trichophyton species-positive suberythrodermic dermatophyte infection. In addition, both the patient and his sister had previously received treatment for ichthyosiform erythroderma [7].

The diversity of etiologies that can result in erythroderma creates a challenge in determining the underlying cause. However, similar to our patient, a skin biopsy may be helpful in elucidating the underlying condition. Indeed, the presence of fungal hyphae in the stratum corneum established that a dermatophyte infection was associated with our patient's chronic erythroderma.

## Conclusions

There are several potential etiologies for erythroderma. The clinical features and course of a man who presented with chronic erythroderma secondary to a dermatophyte infection are described. Although uncommon, tinea corporis is a potential cause for either new-onset or chronic erythroderma; a biopsy of the erythrodermic skin that demonstrates fungal hyphae in the stratum corneum can establish dermatophyte infection as the underlying cause of the erythroderma.
